# 1α,25-Dihydroxyvitamin D Downregulates Adipocyte Impact on Breast Cancer Cell Migration and Adipokine Release

**DOI:** 10.3390/nu16183153

**Published:** 2024-09-18

**Authors:** Chaehyun Yum, Chaylen Andolino, Brienna Larrick, Madeline P. Sheeley, Dorothy Teegarden

**Affiliations:** 1 Interdepartmental Nutrition Program, Department of Nutrition Science, Purdue University, West Lafayette, IN 47907, USA; 2 Institute for Cancer Research, Purdue University, West Lafayette, IN 47907, USA

**Keywords:** 1α,25-dihydroxyvitamin D, breast cancer, adipocyte, conditioned media, migration

## Abstract

Background/Objectives: Excess adiposity is associated with a higher risk of breast cancer metastasis and mortality. Evidence suggests that dietary vitamin D inhibits breast cancer metastasis. However, the mechanistic link between vitamin D’s regulation of adipocyte metabolism and metastasis has not been previously investigated. Therefore, the purpose of these experiments was to examine the effect of the active form of vitamin D, 1α,25-dihydroxyvitamin D (1,25(OH)_2_D), on adipocyte release of bioactive compounds and whether the impact on adipocytes leads to inhibition of breast cancer cell migration, an important step of metastasis. Methods: Differentiated 3T3-L1 adipocytes were treated with 1,25(OH)_2_D for two days, followed by either harvesting the adipocytes or collecting adipocyte-conditioned media without 1,25(OH)_2_D. A transwell migration assay was conducted with vehicle- or 1,25(OH)_2_D-conditioned media. In order to explore the mechanism underlying effects on breast cancer metastatic capability, the mRNA expression of leptin, adiponectin, insulin-like growth factor (IGF-1), interleukin-6 (IL-6), and monocyte chemoattractant protein-1 (MCP-1) was measured in adipocytes following either vehicle or 1,25(OH)_2_D treatment. Results: Conditioned media from 1,25(OH)_2_D-treated adipocytes inhibited the migration of metastatic MDA-MB-231 breast cancer cells compared to conditioned media from vehicle-treated adipocytes. Treatment of adipocytes with 1,25(OH)_2_D decreased mRNA expression of leptin, adiponectin, IGF-1, IL-6, and MCP-1. Consistent with mRNA expression, concentrations of leptin, adiponectin, IGF-1, and IL-6 in adipocyte-conditioned media were decreased with 1,25(OH)_2_D treatment, although MCP-1 remained unchanged. Conclusions: In summary, these results suggest that 1,25(OH)_2_D alters adipocyte secretions to prevent breast cancer metastasis.

## 1. Introduction

Obesity is a growing medical problem worldwide, as more than 69% of US women are overweight or obese according to 2017–2018 NHANES data [1]. It is reported that high body mass index (BMI) is associated with a lower survival rate of breast cancer patients [2]. Obesity is proposed to increase the risk of breast cancer via multiple mechanisms, including increasing proliferation and inflammation, inhibiting differentiation and apoptosis, or altering specific cell signals to modify cancer cell behavior [3]. According to GLOBOCAN 2018, breast cancer is the leading cancer most frequently diagnosed in females worldwide and contributes to the most cancer deaths for women globally [4]. Breast cancer has a survival rate of 99% when metastasis has not occurred, but when the cancer has metastasized to other organs prior to diagnosis, the five-year survival rate falls to 31% [5]. Therefore, focusing efforts on the prevention of breast cancer metastasis in obese individuals is critical.

Adipokine release from adipose tissue [6] is a promising area of research with potential implications for cancer treatment [7]. Mammary epithelial cells can be affected not only by an endocrine signal from whole-body adipose tissue but also potentially by paracrine signaling from surrounding adipocytes [6]. Obesity-associated adipose tissue dysfunction leads to the aberrant production of adipokines that are proposed to be involved in obesity-associated mammary carcinogenesis [8]. In the context of breast cancer, adipokines are particularly relevant because they can influence cancer cell behavior, including proliferation, migration, and invasion [9]. Examples of adipose tissue adipokines include leptin, adiponectin, insulin-like growth factor (IGF-1), interleukin-6 (IL-6), and monocyte chemoattractant protein-1 (MCP-1). Moreover, research indicates that adipocyte-derived lipids also play a role in promoting breast cancer cell migration, particularly from dysfunctional adipocytes that develop in obese states [10]. These findings underscore the complex role of adipose tissue in cancer progression and offer insights for novel treatment strategies. However, despite these insights, the precise mechanisms by which adipocytes interact with breast cancer cells remain poorly understood.

Vitamin D is derived from the diet or synthesized in the human body [11]. In the liver, 25-hydroxylase converts vitamin D to 25-hydroxyvitamin D (25(OH)D), the circulating form of vitamin D, which is considered to be an indicator of vitamin D status. One meta-analysis demonstrated an inverse relationship between serum 25(OH)D and risk of death from breast cancer [12]. Similarly, in prospective data, African American women who have lower 25(OH)D levels have an increased risk of breast cancer compared to those with a higher level of 25(OH)D [13]. Additionally, vitamin D has been reported to exert anti-proliferative and pro-apoptotic effects directly in breast cancer cells, further supporting its role as a preventive agent. Previous research, including ours, indicates that treatment of 1,25(OH)_2_D directly reduced the migratory and invasive potential of the breast cancer cells [14,15]. It is also reported that serum 25(OH)D levels are inversely proportional to adiposity [16,17,18], thus, vitamin D may be an important factor in modulating health outcomes of obese breast cancer patients. In addition, adipose and breast tissue not only express the vitamin D receptor but also 1α-hydroxylase, which locally converts 25(OH)D to the active metabolite 1,25(OH)_2_D [19,20]. Finally, previous studies suggest that 1,25(OH)_2_D regulates various aspects of adipocyte biology, including differentiation, adipogenesis [21], and inflammation [22]. Overall, this evidence highlights that vitamin D metabolites may exert effects on adipose tissue that lead to inhibiting breast cancer; however, their effects in mediating adipokine synthesis and release in adipocytes are not clear.

Although several studies have explored the interaction between adipocytes and breast cancer cells [13,23], the impact of 1,25(OH)_2_D in regulating this interaction potentially through impacts on adipocytes and in the context of breast cancer cell migration has not been studied. The purpose of the current study was to determine if 1,25(OH)_2_D alters the release of adipokines from differentiated adipocytes, which may contribute to reducing metastatic breast cancer cell migration. We hypothesized that 1,25(OH)_2_D alters adipocyte gene expression and release of adipokines from adipocytes, and the impact on the adipocytes reduces breast cancer cell migration. The results of these studies will provide insights into the impact of vitamin D on adipocytes in regulating breast cancer metastasis.

## 2. Materials and Methods

### 2.1. Chemical and Reagents

Dulbecco’s Modification of Eagle’s Medium (DMEM) was obtained from Corning (Manassas, VA, USA). Fetal calf serum was obtained from Thermo Fisher Scientific (Pittsburgh, PA, USA). The bioactive vitamin D metabolite, 1,25(OH)_2_D, was purchased from Biomol (Plymouth Meeting, PA, USA). 1,25(OH)_2_D was first dissolved in 100% ethanol and added to the media to a final ethanol concentration of <0.1%. Fetal bovine serum (FBS), trypsin, and penicillin/streptomycin were obtained from Life Technologies, Gibco-BRL (Rockville, MD, USA). Insulin (bovine), dexamethasone, and 3-isobutyl-1-methylxanthine were obtained from Sigma-Aldrich (St. Louis, MO, USA). Rosiglitazone was obtained from Cayman Chemical (Ann Arbor, MI, USA). Recombinant leptin was purchased from Peprotech (Rocky Hill, NJ, USA).

### 2.2. Cell Culture

MDA-MB-231 cells, regarded as a highly aggressive metastatic human cell line, are derived from a pleural effusion of a female with metastatic breast cancer. Murine metastatic breast cancer 4T1 cells were a gift from Dr. Michael Wendt. MDA-MB-231 and 4T1 cells were cultured in DMEM, 10% fetal bovine serum, 100 units/mL penicillin, and 100 µg/mL streptomycin in a humidified environment at 37 °C with 5% CO_2_. Murine 3T3-L1 fibroblasts were obtained from American Type Culture Collection (ATCC CL-173; Manassas, VA) and cultured in DMEM containing 10% fetal calf serum with 100 units/mL penicillin and 100 µg/mL streptomycin at 37 °C with 5% CO_2_.

3T3-L1 preadipocytes were differentiated into mature adipocytes following protocols previously described [24]. Cells were grown to confluence (designated as day −2). On day 0, cells were treated with the differentiation cocktail (1 µg/mL insulin, 0.5 mM isobutylmethylxanthine, 1 µM dexamethasone, and 2 µM rosiglitazone) in 10% FBS-containing DMEM. On day 2, the medium was replaced with DMEM containing 1 µg/mL insulin and 10% FCS. On day 4, the medium was changed to 10% FCS-containing DMEM, and the medium was replaced every 2 days. On day 9, differentiated mature adipocytes were treated with vehicle (final ethanol concentration of < 0.1% in medium) or 1,25(OH)_2_D (10 nM for 48 h. After 48 h of treatment, the medium was aspirated, cells were washed with phosphate-buffered saline (pH = 7.4, 137 mM NaCl, 2.7 mM KCl, 10 mM Na_2_HPO_4_, 1.8 mM KH_2_PO_4_) to remove any remaining 1,25(OH)_2_D present, and then incubated for another 24 h in 10% FBS-containing DMEM. The media was collected, sterile-filtered to remove cellular debris, and stored at −20 °C until use.

### 2.3. Migration Assay

Differentiated mature adipocytes were treated with vehicle or 1,25(OH)_2_D (10 nM) for two days. After 2 d of treatment, the medium was replaced with 10% FBS-containing DMEM for 24 h and was then collected (denotated as “adipocyte-conditioned media”). MDA-MB-231 cells were plated in serum-free medium into 8 µm pore FluoblockTM-coated Boyden chamber transwell inserts. Transwell inserts were placed into adipocyte-conditioned media treated either with vehicle or 1,25(OH)_2_D. Additionally, negative control samples of the cells were plated as above and placed inside a well containing serum-free media. After 15 h of incubation, transwell inserts were removed and placed in dark-coated 24-well plates containing 2.5 ug/mL Calcein AM in 1× clear PBS. Migration was quantified after incubation using a bottom fluorescent plate reader (Ex./Em. 495/525). Results were normalized to the average of the negative control samples and expressed as a fold change relative to the conditioned medium from vehicle-treated adipocytes.

### 2.4. Analysis of Concentration of Adipokines

The leptin and IGF-1 levels of conditioned media were measured by a mouse enzyme-linked immunosorbent assay (ELISA) kit (RayBiotech, Peachtree Corners, GA, USA). The level of adiponectin in conditioned media was assessed using an ELISA kit (R&D Systems, Minneapolis, MN, USA). The level of IL-6 in conditioned media was assessed using a V-plex assay (Meso Scale Diagnostics LLC, Rockville, MD, USA). The level of MCP in conditioned media was measured by CCL2 MCP-1 ELISA (Invitrogen, Carlsbad, CA, USA).

### 2.5. RNA Isolation and Analysis

Adipocytes were lysed using TriReagent (Molecular Research Center, Cincinnati, OH, USA) for RNA isolation, and reverse transcription to cDNA was conducted using MMLV reverse transcriptase (Promega, Madison, WI, USA) as previously described [25]. mRNA expression was determined using qPCR and data normalized to 18S ribosomal RNA (18S) expression and using the comparative Ct method (2^−ΔΔCt^). Data are expressed as fold change relative to the vehicle. Primers used are shown in Table 1.

### 2.6. Glycerol Release Assay

Adipocyte-conditioned media samples from adipocytes were used for the analysis of glycerol. Glycerol was quantified using a spectrophotometric assay kit from Sigma-Aldrich (St. Louis, MO, USA).

### 2.7. Statistical Analysis

Values are presented as mean ± SEM. Results are expressed compared to the vehicle control sample by Student’s *t*-tests, with *p* < 0.05 considered statistically significant.

## 3. Results

**Figure 1:**
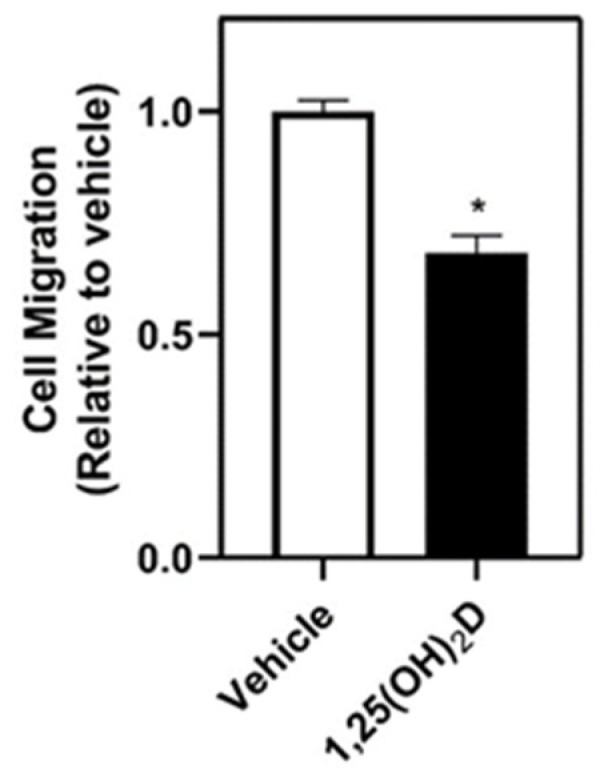
The effect of adipocyte-conditioned media from 1,25(OH)_2_D-treated adipocytes on migration in MDA-MB-231 breast cancer cells. Adipocyte-conditioned media collected from differentiated 3T3-L1 adipocytes that were treated with vehicle or 1,25(OH)_2_D (10 nM) was used to determine the impact on migration of MDA-MB-231 cells employing a transwell assay. An asterisk indicates a significant difference relative to vehicle control (*p* < 0.05).

We examined the impact of 1,25(OH)_2_D-mediated regulation of differentiated adipocytes on breast cancer cell migration. Adipocyte-conditioned media from 1,25(OH)_2_D-treated adipocytes, collected 24 h after replacing the media with media without 1,25(OH)_2_D, placed in the bottom well reduced MDA-MB-231 cell migration compared to conditioned media from vehicle-treated adipocytes (Figure 1), suggesting that 1,25(OH)_2_D reduces the chemoattractive effect of adipocytes. Therefore, we sought to identify factors released from adipocytes that may impact chemoattraction that are altered following 1,25(OH)_2_D treatment.

**Figure 2:**
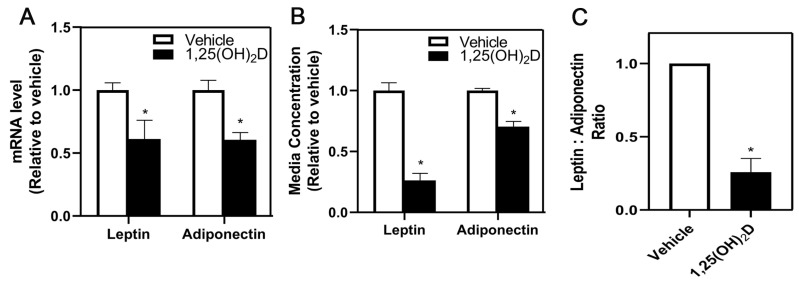
mRNA levels and release of adipokines, leptin, and adiponectin following vehicle or 1,25(OH)_2_D treatment of mature 3T3-L1 adipocytes. (**A**) Leptin and adiponectin mRNA levels were measured following vehicle or 1,25(OH)_2_D treatment in adipocytes by reverse-transcription PCR and normalized to 18S. (**B**) The concentration of leptin and adiponectin in conditioned media from 3T3-L1 adipocytes following treatment of vehicle or 1,25(OH)_2_D was measured. (**C**) Leptin/adiponectin ratio was calculated from results shown in (**B**). The values are expressed per vehicle and as mean ± SEM. An asterisk indicates a significant difference relative to the vehicle (*p* < 0.05).

Because leptin and adiponectin are secreted from adipocytes and have been previously shown to alter metastasis [26], the impact of 1,25(OH)_2_D on the expression and release of these adipokines was assessed. 1,25(OH)_2_D decreased leptin and adiponectin mRNA expression in differentiated 3T3-L1 adipocytes compared to vehicle-treated adipocytes (Figure 2A). The concentration of leptin and adiponectin was measured in the adipocyte-conditioned media to determine if the decrease in gene expression is associated with a similar decrease in the level of adipokines in the media. Consistent with the mRNA results, 1,25(OH)_2_D treatment decreased protein concentration of leptin and adiponectin in adipocyte-conditioned media compared to vehicle treatment (Figure 2B). The leptin/adiponectin ratio is suggested to be altered in obesity [27], therefore we measured these proteins in adipocyte-conditioned media. Interestingly, 1,25(OH)_2_D decreased the leptin/adiponectin ratio in adipocyte-conditioned media compared to vehicle treatment (Figure 2C).

**Figure 3:**
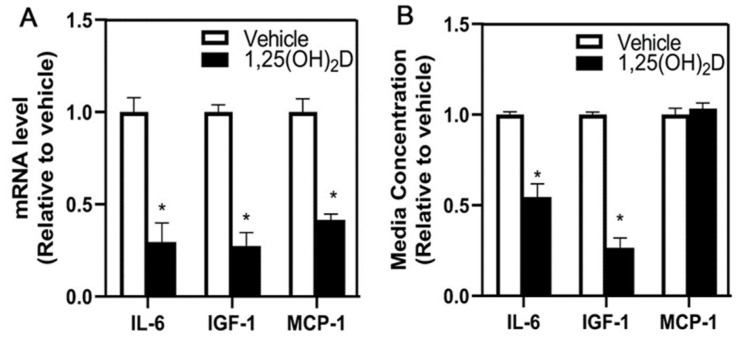
mRNA levels and release of adipokines, IL-6, IGF-1, and MCP-1 following vehicle or 1,25(OH)_2_D treatment of mature 3T3-L1 adipocytes. (**A**) IL-6, IGF-1, and MCP-1 mRNA levels were measured following vehicle or 1,25(OH)_2_D treatment in adipocytes by reverse-transcription PCR and normalized to 18S. (**B**) The concentration of IL-6, IGF-1, and MCP-1 in conditioned media from 3T3-L1 with treatment of vehicle or 1,25(OH)_2_D was measured. The values are expressed per vehicle and as mean ± SEM. An asterisk indicates a significant difference relative to the vehicle (*p* < 0.05).

To explore other potential factors that are altered in adipocytes following 1,25(OH)_2_D treatment, we measured mRNA expression of IL-6, IGF-1, and the chemokine MCP-1. Treatment of adipocytes with 1,25(OH)_2_D decreased mRNA expression of IL-6, IGF-1, and MCP-1 (Figure 3A). The media concentration was assessed to further explore if the decreased mRNA levels corresponded to an altered release of these factors. 1,25(OH)_2_D treatment of adipocytes reduced the media concentration of IL-6 and IGF-1, but MCP-1 concentration remained unchanged by 1,25(OH)_2_D treatment (Figure 3B), suggesting that MCP-1 is not a factor required to reduce the migration of MDA-MB-231 cells by 1,25(OH)_2_D-treated adipocyte-conditioned media.

**Figure 4:**
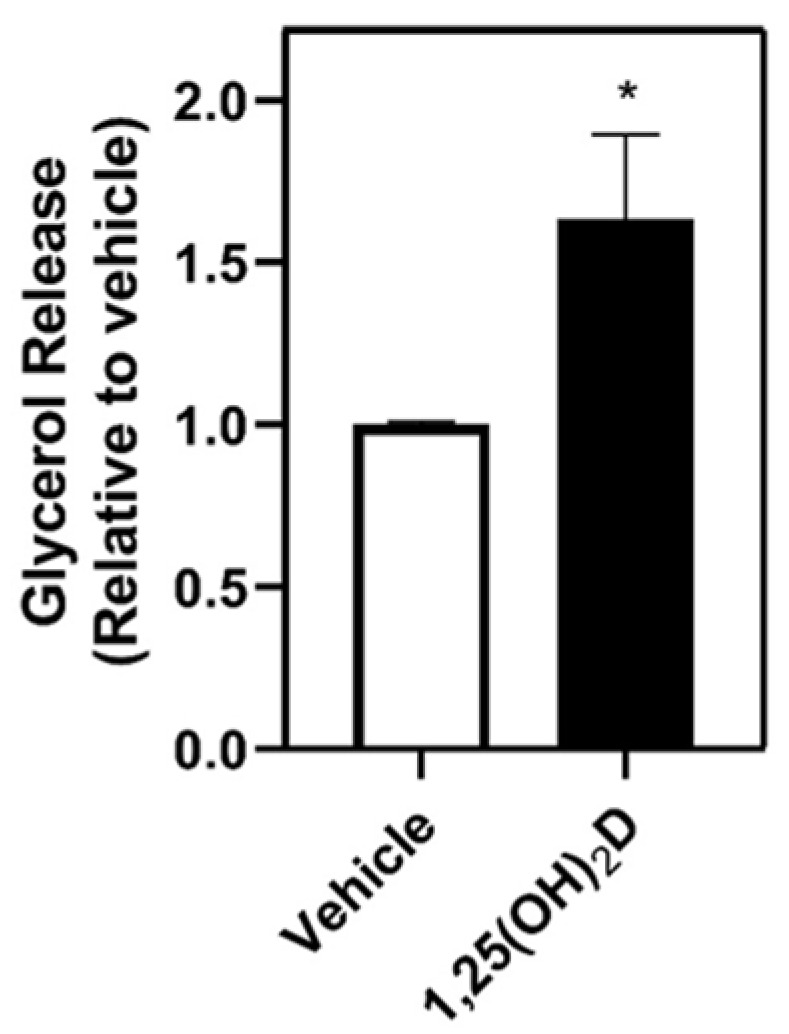
Glycerol release following 1,25(OH)_2_D treatment in mature 3T3-L1 adipocyte. Differentiated 3T3-L1 adipocytes were treated with vehicle or 1,25(OH)_2_D (10 nM) for 48 h. Glycerol was measured in adipocyte-conditioned media by enzymatic determination of free glycerol. The values are expressed per vehicle and as mean ± SEM. An asterisk indicates a significant difference relative to vehicle control (*p* < 0.05).

Previous research indicates that lipids produced by adipocytes play a role in enhancing the migratory properties of breast cancer cells [28]. To address whether 1,25(OH)_2_D impacts the hydrolysis of adipocyte triacylglycerol, we measured the lipolytic product glycerol in the media. Treatment of 1,25(OH)_2_D for two days increased glycerol release in mature adipocytes (Figure 4), indicating 1,25(OH)_2_D may increase triacylglycerol hydrolysis.

## 4. Discussions

It is reported that weight gain following menopause elevates the risk of breast cancer in postmenopausal women [29]. Similarly, a meta-analysis shows that high BMI is associated with lower survival in pre- and post-menopausal breast cancer patients [2], and Mohr et al. have demonstrated that serum 25(OH)D level is inversely correlated to the risk of breast cancer-related death [12]. Evidence also suggests that vitamin D status is negatively associated with obesity [16,17], potentially due to the fact that vitamin D could become sequestered within the excess adipose tissue. Given that there are known impacts of vitamin D on adipocyte metabolism [24], these alterations in the adipocyte may indirectly impact the progression of breast cancer. In this study, we demonstrated that adipocyte-conditioned media from 1,25(OH)_2_D-treated adipocytes significantly inhibited the migration of MDA-MB-231 breast cancer cells. We confirmed that 1,25(OH)_2_D inhibited the release of adipokines, including proinflammatory cytokines and chemokines, from adipocytes previously implicated in increasing migratory capability in breast cancer cells. These results suggest that there may be multiple synergistic mechanisms by which 1,25(OH)_2_D contributes to the inhibition of breast cancer cell migration.

Adipose tissue secretes adipokines such as leptin and adiponectin. Leptin is secreted from adipose tissues in proportion to the size of adipocytes [30]. Despite being produced by adipocytes, adiponectin levels in the bloodstream typically show an inverse relationship with body fat content [31]. Studies show that obese individuals have lower circulating adiponectin concentrations compared to their non-obese counterparts [31]. Emerging evidence suggests that elevated leptin levels promote breast cancer progression. A meta-analysis conducted by Pan et al. demonstrated an association between elevated serum leptin levels and the progression of breast cancer [32]. Furthermore, treatment of MDA-MB-231 breast cancer cells with 50 µM of leptin increased migration [33], and leptin administration increases the primary tumor and the metastasis to the lung of breast tumor cells in vivo [34]. In our study, 1,25(OH)_2_D reduced the concentration of leptin present within conditioned media from adipocytes, suggesting that leptin may mediate the impact of 1,25(OH)_2_D on adipocytes to reduce breast cancer cell migration.

Adipose tissue produces and releases inflammatory cytokines, which play a role in obesity-related inflammation. This chronic inflammatory state has been associated with a higher risk of breast cancer development in people with obesity. Proinflammatory cytokine IL-6 is upregulated in obesity [35] and has also been shown to increase breast cancer cell migration [36]. In our study, 1,25(OH)_2_D treatment decreased adipocyte mRNA expression and IL-6 release into the adipocyte media. This finding is consistent with the results of Chang et al., which demonstrated that vitamin D insufficiency significantly elevated IL-6 levels in both adipose tissue and serum of high-fat diet-induced obese rats [37]. These observations suggest a potential role for 1,25(OH)_2_D in modulating obesity-related inflammation and potentially breast cancer migration.

Furthermore, 1,25(OH)2D treatment decreased both mRNA expression and release of IGF-1 from mature 3T3-L1 adipocytes in the current study. Previous research has established a connection between elevated IGF-1 levels and obesity [38], as well as a link between IGF-1 and a higher risk of developing breast cancer [39]. Thus, a decrease in IGF-1 from adipocytes mediated by 1,25(OH)_2_D treatment may play a role in decreasing breast cancer cell migration. However, the role of vitamin D in regulating IGF-1 levels is not entirely clear, as other studies have shown mixed results. For instance, one randomized controlled trial by Trummer et al. reported no change in IGF-1 concentrations with vitamin D supplementation in hypertensive patients with low 25(OH)D levels [40], while Kamycheva et al. reported a reduction in the IGF-1/IGF binding protein-3 ratio only in individuals with a BMI below 35 kg/m^2^ [41]. These discrepancies may be due to differences in study design and participant characteristics; therefore, further research is necessary to clarify the role of vitamin D in regulating IGF-1 levels in obese individuals and to explore the independent role of IGF-1 in breast cancer cell migration. Furthermore, studies have demonstrated that MCP-1 acts as a chemotactic factor, promoting the movement of breast cancer cells [42]. Even though 1,25(OH)_2_D also decreases MCP-1 mRNA expression in adipocytes, the level of MCP-1 protein in conditioned media was not changed following 1,25(OH)_2_D treatment of adipocytes, indicating that this factor is likely not involved in the 1,25(OH)_2_D-mediated reduction in migration from adipocyte-conditioned media observed in our studies.

The complex interplay between various adipokines from adipocytes creates a multifactorial influence on breast cancer cell migration. For instance, while leptin may directly enhance cell migration, its impact could be modulated by other factors such as IL-6 or IGF-1, which are also upregulated in obesity. The precise effect of each factor will vary depending on the overall composition and environment of the adipose tissue, as well as the characteristics of the breast cancer cells. This complex network of interactions suggests that when studying breast cancer progression, it is important to examine the combined effects of adipokines and cytokines rather than isolated impacts.

Another potential mechanism by which 1,25(OH)_2_D regulation of adipocytes decreases breast cancer cell migration may be via altered hydrolysis of triacylglycerol, thereby leading to a change in the release of glycerol. Interestingly, research findings indicated that glycerol enhanced the progression of prostate cancer in mice implanted with human tumor cells [43]. In our study, 1,25(OH)_2_D treatment increased glycerol release by mature adipocytes, suggesting that 1,25(OH)_2_D may increase lipolysis. Larrick et al. also showed that 1,25(OH)_2_D treatment increases glycerol release by increasing phosphorylation of hormone-sensitive lipase without the release of fatty acids into the media from adipocytes [21]. This suggests either an increase in glyceroneogenesis, intracellular reutilization of the fatty acids, or efficient reuptake of fatty acids into the adipocytes.

Our study is the first to investigate the impact of 1,25(OH)_2_D-mediated adipocyte-conditioned media on breast cancer migratory capability. The results of the current study confirm that 1,25(OH)_2_D inhibits the migratory potential of breast cancer cells and that this may be due in part to an indirect mechanism—by altering adipocyte release of chemoattractants, such as leptin, adiponectin, IL-6, and IGF-1. It is possible that one or several of these factors may function together to alter breast cancer cell migration. It is of interest to investigate the impact of 1,25(OH)_2_D-treated adipocyte-conditioned media on the migration of additional breast cell lines. These findings support the potential use of vitamin D in preventing breast cancer progression, particularly by reducing the propensity of breast cancer cells to migrate.

## Figures and Tables

**Table 1:** Primers used in the qPCR analysis of gene expression.

Genes	Forward 5′-3′	Reverse 5′-3′
*Adiponectin*	ACCAAAAGGGCTCAGGATGC	GAGCGATACACATAAGCGGC
*Igf-1*	TGGATGCTCTTCAGTTCGTG	TTTTGTAGGCTTCAGTGGGG
*Il-6*	AGTGGCTAAGGACCAAGACC	TCTGACCACAGTGAGGAATG
*Lep*	GCAAGAAGAAGAAGATCCCAGG	CAGATAGGACCAAAGCCACAG
*Mcp-1*	GCAGCAGGTGTCCCAAAGAA	ATTTAGGGTCAACTTCACATTCAA
*18S*	ATCCCTGAGAAGTTCCAGCA	CCTCTTGGTGAGGTCGATGT

## Data Availability

All of the data are contained within the article.

## Abbreviations

1α:25-dihydroxyvitamin D—1,25(OH)_2_D; insulin-like growth factor—IGF-1; interleukin-6—IL-6; monocyte chemoattractant protein-1—MCP-1.
